# Characteristics of splenic PD-1^+^ γδT cells in *Plasmodium yoelii nigeriensis* infection

**DOI:** 10.1007/s12026-023-09441-w

**Published:** 2024-01-24

**Authors:** Dianhui Chen, Feng Mo, Meiling Liu, Lin Liu, Junmin Xing, Wei Xiao, Yumei Gong, Shanni Tang, Zhengrong Tan, Guikuan Liang, Hongyan Xie, Jun Huang, Juan Shen, Xingfei Pan

**Affiliations:** 1 Department of Infectious Diseases, Guangdong Provincial Key Laboratory of Major Obstetric Diseases; Guangdong Provincial Clinical Research Center for Obstetrics and Gynecology; The Third Affiliated Hospital of Guangzhou Medical University, Guangzhou, China; 2 Clinical Laboratory, The Third Affiliated Hospital of Guangzhou Medical University, Guangzhou, 510150 China; 3 China Sino-French Hoffmann Institute, Department of basic Medical Science, Guangzhou Medical University, Guangzhou, 511436 China; 4 State Key Laboratory of Respiratory Disease, National Clinical Research Center for Respiratory Disease, Guangzhou, China; 5 Kingmed School of Laboratory Medicine, Guangzhou Medical University, Guangzhou, Guangdong 510182 People’s Republic of China

**Keywords:** *Plasmodium yoelii nigeriensis* NSM, PD-1, γδT cells, RORα, NF-κB

## Abstract

Although the functions of programmed death-1 (PD-1) on αβ T cells have been extensively reported, a role for PD-1 in regulating γδT cell function is only beginning to emerge. Here, we investigated the phenotypic and functional characteristics of PD-1-expressing γδT cells, and the molecular mechanism was also explored in the *Plasmodium yoelii nigeriensis* (*P. yoelii* NSM)-infected mice*.* Flow cytometry and single-cell RNA sequencing (scRNA-seq) were performed. An inverse agonist of RORα, SR3335, was used to investigate the role of RORα in regulating PD-1^+^ γδT cells. The results indicated that γδT cells continuously upregulated PD-1 expression during the infection period. Higher levels of CD94, IL-10, CX3CR1, and CD107a; and lower levels of CD25, CD69, and CD127 were found in PD-1^+^ γδT cells from infected mice than in PD-1^−^ γδT cells. Furthermore, GO enrichment analysis revealed that the marker genes in PD-1^+^ γδT cells were involved in autophagy and processes utilizing autophagic mechanisms. ScRNA-seq results showed that RORα was increased significantly in PD-1^+^ γδT cells. GSEA identified that RORα was mainly involved in the regulation of I-kappaB kinase/NF-κB signaling and the positive regulation of cytokine production. Consistent with this, PD-1-expressing γδT cells upregulated RORα following *Plasmodium yoelii* infection. Additionally, in vitro studies revealed that higher levels of p-p65 were found in PD-1^+^ γδT cells after treatment with a RORα selective synthetic inhibitor. Collectively, these data suggest that RORα-mediated attenuation of NF-κB signaling may be fundamental for PD-1-expressing γδT cells to modulate host immune responses in the spleen of *Plasmodium yoelii nigeriensis*–infected C57BL/6 mice, and it requires further investigation.

## Introduction

Malaria is typically transmitted to humans by the bite of a female Anopheles mosquito, the carrier of protozoan parasites of the genus *Plasmodium* [1]. The mortality rate from malaria is approximately 0.26% worldwide, mostly caused by *Plasmodium falciparum* [2, 3]. Five species of Plasmodia are known to cause disease in humans, including *Plasmodium falciparum*, *Plasmodium ovale*, *Plasmodium malariae*, *Plasmodium vivax* and *Plasmodium knowles* [4]. Rodent malaria parasites, *Plasmodium berghei* and *Plasmodium yoelii*, may have advantages in stable transformation and gene targeting [5, 6]. They have become key model systems for studying the basic biology of malaria parasites. The life cycle of *Plasmodium* in the host comprises two stages in two different tissues, the liver and blood. Infective sporozoites migrate in the skin, enter blood vessels, and from there, find their way to the liver [7]. A single sporozoite can mature into thousands of merozoites. These merozoites are released into the bloodstream where they invade red blood cells and initiate the blood-stage infection [8]. The development of malaria is facilitated by a variety of immune mechanisms, such as the production of anti- or proinflammatory cytokines, induction of immune cells, and production of IgG antibodies [9, 10].

T cells are subdivided into two large populations distinguished by their surface expression of αβ and γδT-cell receptors (TCRs) [11]. Compared to traditional CD4^+^ and CD8^+^ alpha beta (αβ) T cells, γδT cells display extensive functional plasticities, such as antigen-presenting capacity and B-cell helper activity, and have the potential for pro- and anti-inflammatory cytokines production. γδT cells constitute approximately 5–10% of the circulating T-cell population, which can be found in the blood and lymphoid organs or as resident cells in peripheral tissues and barrier surfaces [12]. It was reported that γδT cells might be more effective than monocyte-derived dendritic cells at cross-presentation in vitro [13]. Extensive evidence shows that γδT cells are cytotoxically active and produce cytokines associated with both protective immunity and symptomatic episodes during human malaria infection [14, 15].

Immune checkpoint molecules, programmed cell death-1 (PD-1), lymphocyte activation gene-3 (LAG-3), T-cell immunoglobulin and ITIM domain (TIGIT), and T-cell immunoglobulin-3 (TIM-3) are inhibitory receptors expressed on immune cells, such as T cells, B cells, and certain myeloid cells [16]. Studies have also shown that vaccines have not been successful because of apoptosis of vaccine-specific memory B cells and several other factors [17, 18]. The role of PD-1 as a major factor in the loss of immunity against malaria has risen to the forefront [19]. In general, PD-1 expression on T cells is induced by antigen stimulation that can trigger immunosuppressive signaling pathways [19]. A deeper understanding of the interplay between immune checkpoints and their ligands, which is complex and occurs at different phases of T-cell activation and function, is needed [20]. Several reports have also provided strong evidence that sustained antigenic stimulation or an immunosuppressive microenvironment maintains high PD-1 and LAG-3 expression on T-cells and exhibits an “exhausted” or dysfunctional phenotype, which prevents robust host protective effector T-cell responses in the context of chronic viral infections and tumors [21, 22]. PD-1 is upregulated in various nonlethal mouse models of blood-stage malaria [23, 24]. Aside from PD-1, TIM-3 may also contribute to T-cell exhaustion. In malaria infection, TIM-3 upregulation attenuated Vδ2 T-cell responses [15]. PD-1 and LAG-3 are highly expressed in CD4^+^ and CD8^+^ T cells, and the expansion of such cells among peripheral blood mononuclear cells in malaria-infected patients has been reported [25]. Consistent with this finding, our previous study found that the proportions of PD-1^+^ CD4^+^ T cells increased post-*P. yoelii* infection and it appeared to be more activated and could secrete more cytokines to modulate host immune responses [26]. In murine malaria models, PD-1, TIM-3, and LAG-3 blockade leads to an enhancement of pro-inflammatory T-cell responses and a more severe course of disease. However, it can also improve parasite clearance.

T-cell exhaustion is regulated by distinct transcription factors, including TOX, PTPN2, TCF-1, and Eomes [27–29]. Furthermore, it was reported that antigen stimulation also leads to sustained expression of PD1 through NFAT cytoplasmic 1 (NFATc1), signal transducer and activator of transcription 3 (STAT3), IFN-stimulated gene factor 3 (ISGF3), and so on [30, 31]. Mouse Eomes^hi^ PD-1^hi^ γδT cells coexpressed Th1 lineage-related factors such as CD27, Ly6C, and T-bet were less capable of IFN-γ production, indicating that Eomes is a marker for the differentiation exhaustion of Th1-like effector γδT cells [32]. Furthermore, it was reported that NFATc1 and PD-1 expression could be induced in CD4 T cells through HIF-1a after *P. yoelii* infection [26]. The expression of PD-1 on CD4^+^ and CD8^+^ T cells has been reported to provide modulatory signals during malaria, but its function in the modulation of γδT cells remains unresolved. Hence, this study will primarily focus on the function and mechanism of PD-1-expressing γδT cells in the spleen of C57BL/6 mice infected by *P. yoelii NSM*.

## Materials and methods

### Mice

Female C57BL/6 mice were purchased from Traditional Chinese Medicine University of Guangzhou Animal Center (Guangzhou, China) at 6–8 weeks of age. All animal experiments were reviewed and performed in strict accordance with the Regulations for the Administration of Affairs Concerning Experimental Animals (1988.11.1). The animal protocols were approved to be appropriate and humane by the institutional animal care and use committee of Guangzhou Medical University (2012-11).

### Infection

The NSM strain of *Plasmodium yoelii* was obtained from MR4 (Malaria Research and Reference Reagent Resource Center). Frozen *P. yoelii* were thawed at 37 °C in a water bath. They were maintained in mice by intraperitoneal injection, and parasitemia was monitored daily up to 10–15%. Mice were infected with 1 × 10^6^ infected red blood cells (iRBCs) by intraperitoneal injection. Moreover, 28 mice were infected for dynamic observation of PD-1 expression. At 4, 8, 12, 16, 20, 24, and 28 days post-infection, four mice were randomly chosen and sacrificed. Four pathogen-free mice constituted the control group.

### Reagents and antibodies

APC- conjugated anti-mouse CD45 (30-F11), APC-cy7-conjugated anti-mouse CD3 (145-2C11), FITC-conjugated anti-mouse CD3 (17A2), FITC-conjugated anti-mouse γδ TCR (B1), BV510-conjugated anti-mouse γδ TCR (GL3), PerCP-cy5.5-conjugated anti-mouse CD4 (RM4-5), PE-conjugated anti-mouse CD8 (53-6.7), PE-cy7-conjugated anti-mouse PD-1 (29F.1A12), PE- conjugated anti-mouse CD25 (BC96), APC-conjugated anti-mouse CD69 (H1.2F3), PE-conjugated anti-mouse CD44 (QA19A43), PE-conjugated anti-mouse CD94 (18d3), APC- conjugated anti-mouse CD62L (MEL-14), APC-conjugated anti-mouse CD314(CX5), APC-conjugated anti-mouse IFN-γ(XMG1.2), PE-conjugated anti-mouse IL-10 (JES5-16E3), BV421-conjugated anti-mouse CXCR5 (L138D7), FITC-conjugated anti-mouse CX3CR1(SA011F11), Alexa Fluor® 488 anti-mouse CD107a(1D4B), PE/Cyanine7 anti-mouse TIGIT(1G9), and APC anti-mouse CD223(C9B7W) were purchased from BioLegend (San Diego, CA, USA) and BD Pharmingen (San Diego, CA, USA). SR3335 (HY-14413) was purchased from MedChemExpress.

### Lymphocyte isolation

Mice were euthanized at 12–14 days after infection. The spleen tissue was pressed through a 200-gauge stainless-steel mesh and suspended in Hank’s balanced salt solution. The cell suspension was treated with RBC lysis buffer for 5 mins. Then, the isolated cells were washed twice with HBSS (Hank’s balanced salt solution). Finally, cells were resuspended at 2 × 10^6^ cells/ml in a complete RPMI 1640 medium. The complete RPMI 1640 medium supplemented with 10% heat-inactivated fetal calf serum, 100 U/ml penicillin, 100 mg/ml streptomycin, 2 mM glutamine, and 50 mM 2-mercaptoethanol.

### Flow cytometry (FCM) analysis

Cells were washed in PBS and then stained with conjugated antibodies specific for the cell surface antigens for 30 min at 4 °C in the dark. The expression phenotypes of antibody-labeled cells were analyzed by flow cytometry (Beckman CytoFLEX). Isotype-matched controls for cell surface markers were included in each staining protocol. For intracellular cytokine staining, cells were then stimulated with phorbol 12-myristate 13-acetate (PMA) (20 ng/ml, Sigma–Aldrich, St. Louis, MO) and ionomycin (1 μg/ml, Sigma–Aldrich, St. Louis, MO) for 5 hr at 37 °C under a 5% CO_2_ atmosphere. Brefeldin A (10 μg/ml, Sigma–Aldrich, St. Louis, MO) was added during the last 4 h of incubation to stop the stimulation. Cells were washed in PBS and stained for 30 min at 4 °C in the dark with conjugated antibodies specific for the cell surface antigens. After that, the cells were fixed and permeabilized with a Fixation/Permeabilization Solution Kit (555028, BD Biosciences) for 20 min at 4 °C in the dark. Then, the cells were stained with conjugated antibodies specific for cytokines. Expression phenotypes of antibody-labeled lymphocytes were analyzed using flow cytometry (Beckman CytoFLEX), and the results were analyzed using CytExpert 1.1 (Beckman Coulter Inc.).

### 10 × Genomics Chromium library construction and sequencing

Mice were sacrificed, and spleens were collected from three infected and three naïve mice, respectively. Single-cell solutions were prepared, and CD3^+^γδ TCR^+^ γδT cells were sorted by FACS (MoFlo XDP). The expression of RNA in each cell was detected by 10 × Genomics Chromium Single Cell RNA Sequencing (sc-RNA-seq, LC-biotechnology, LTD, Hangzhou, China). The GemCode™ TM Single Cell platform (10 × Genomics, Pleasanton, CA) was used to determine the transcriptomes of single cells. Fifteen microliters of single-cell suspension at a concentration of ~900,000 cells/ml was loaded into one channel of the ChromiumTM Single Cell G Chip (10 × Genomics, 1000073), aiming for a recovery of 8000–9000 cells. The Chromium Single Cell 3′ Library & Gel Bead Kit v3 (10 × Genomics, 1000075) was used for single-cell barcoding, cDNA synthesis, and library preparation, following the manufacturer’s instructions according to the Single Cell 3′ Reagent Kit User Guide Version 3. Libraries were sequenced on an Illumina NovaSeq6000 using paired-end 150 bp.

### scRNA-seq data processing, quality control, and filtering

CellRanger (version 5.0.1) was used to align reads on the GRCm38 reference genome for mice and generate unique molecular identifier gene expression profiles for every single cell under a standard sequencing quality threshold (default parameters). Low-quality cells were removed for downstream analysis when they met the following criteria for retaining cells: (1) ≥ 50,000 sequence reads; (2) ≥ 40% of reads uniquely aligned to the genome; and (3) ≥40% of these reads mapped to RefSeq annotated exons. The Seurat (Version 4.1.0) R package was used to process the UMI counts mentioned above with further filtering criteria (cells were removed): (1) less than 200 and more than 6000 expressed genes, (2) higher than 10% and 10% mitochondrial genome transcripts, (3) fewer than 3 cells, and (4) more than 50,000 UMI counts. Eventually, 28,846 cells and 17,730 genes were retained in the data.

### Normalization, scaling, and clustering

The final filtered gene expression data matrix was normalized using the “NormalizeData” function with the default setting. We chose 3000 highly variable genes via the “FindVariableGenes” function from the final filtered count matrix and then centered and scaled them via the “ScaleData” function. Principal component analysis (PCA) of the 3000 genes was then performed by the “RunPCA” function. Dimensional reduction was performed through canonical correlation analysis (CCA) in Seurat. Cells were clustered by the “FindClusters” function, and the clustered cells were then projected onto a two-dimensional space using the “RunT-SNE” function. The clustering results were visualized by the “DimPlot” function. Identification of differentially expressed genes (DEGs) and enrichment analysis: DEGs were identified by the “FindMarkers” function in Seurat using “wilcox” test methods and Bonferroni correction. Significant DEGs were selected from genes with an adjusted *P*-value (*p*_val_adj) ≤ 0.05 and log processed average fold change (avg_log_2_FC) ≥ 0.25 for further analysis and visualization. GO analysis and KEGG pathway enrichment analysis of these significant DEGs were performed by the clusterProfiler package.

### The measurement of NF-κB regulation by RORα

Mice were infected with *P. yoelii* 12 to 14 days later, and the spleens were removed. Single cells were treated with SR3335 (HY-14413, MedChemExpress) at doses of 5, 10, and 20 μM for 2 days. Anti-CD3 mAb (1 μg/ml) and anti-CD28 mAb (1 μg/ml) were added to each well. Then, the level of NF-κB was measured by flow cytometry (Beckman CytoFLEX).

### Statistics

Data were analyzed by SPSS 21.0, and statistical evaluation of the difference between means was assessed using one-way ANOVA. *P* < 0.05 was considered to be statistically significant.

## Results

### *P. yoelii* infection could increase the percentage of PD-1^+^ γδT cells in the spleen of C57BL/6 mice

**Fig. 1:**
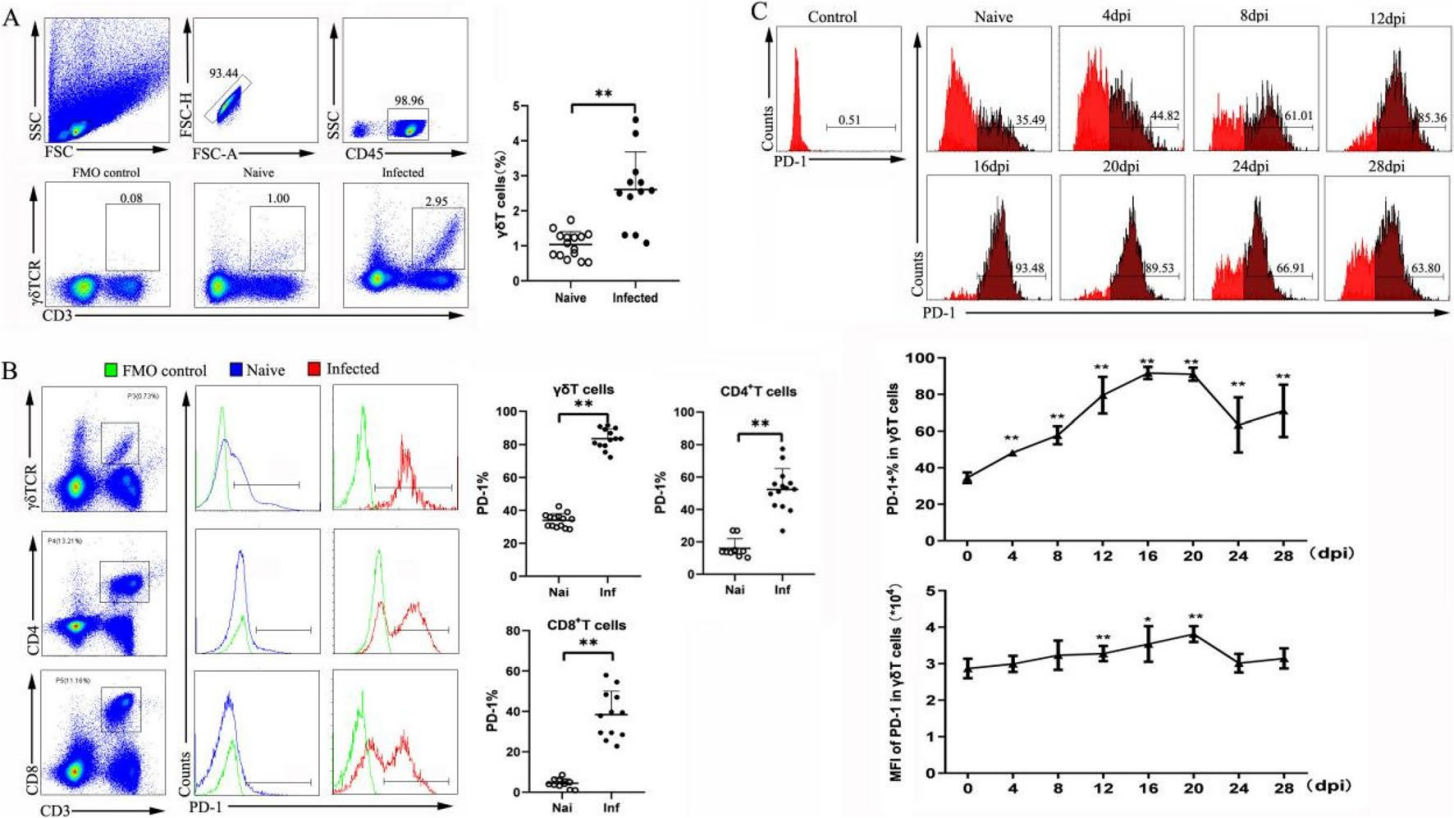
The expression of PD-1 in γδT cells increased upon *P. yoelii* NSM infection. **A** Splenic lymphocytes were stained with anti-CD3 and anti-γδTCR fluorescent mAbs. The expression of CD3 and γδT on spleen lymphocytes of naive and infected mice was analyzed by flow cytometry. Flow cytometric analysis from one representative experiment and average percentages of γδT cells were calculated. **B** PD-1 expression by gated populations of γδT cells, CD4^+^ T cells, and CD8^+^T cells from normal and infected mice, respectively. The proportions of PD-1^+^γδT cells, PD-1^+^CD4^+^T cells, and PD-1^+^CD8^+^T cells from naive and infected mice were compared. **C** The dynamic changes of PD-1^+^ γδT cells in *P. yoelii* NSM-infected mice were investigated from 0 to 28 days. A total of 3–5 samples were prepared for each group, and the experiments were repeated three times. One million cells from one animal were stained for the cell surface antigens. *P < 0.05, **P < 0.01, the error bars indicate SD

To examine the expression of PD-1 in the spleens of *Plasmodium yoelii*–infected mice, the mice were euthanized 12–14 days post-infection, and spleens were removed. Single-cell suspensions were prepared and calculated with trypan blue staining. First, the percentage of CD3^+^ γδTCR^+^ cells was determined by flow cytometry (Fig. 1A). All doublet cells and nonlymphoid cells were excluded from this study. The percentage of γδT cells in the infected group was significantly higher than that in the naïve group (*P* < 0.01). Interestingly, the percentage of PD-1-expressing γδT cells in the infected mice was higher than that in the naïve mice (83.65 ± 6.06% versus 33.99 ± 4.14%, *P* < 0.01, Fig. 1B). Compared with the naïve group, the proportions of PD-1^+^ CD4^+^ T cells and PD-1^+^ CD8^+^ T cells also increased significantly during *P. yoelii* infection (CD4^+^, 83.65 ± 6.06% versus 33.99 ± 4.14%, *P* < 0.01; CD8^+^, 4.43 ± 2.22% versus 38.40 ± 11.61%, *P* < 0.01). Then, the dynamic changes in PD-1 expression on γδT cells were investigated in *P. yoelii*–infected mice (Fig. 1C). The percentage of PD-1^+^ γδT cells markedly increased with the prolongation of infection and peaked in the middle of infection. After a slight decrease at 24 days, the percentage continued to increase at day 28 post-infection. Similarly, the mean fluorescence intensity (MFI) of PD-1 on γδT cells was significantly increased, and then the percentages gradually decreased at 24 days. These results suggested that PD-1^+^ γδT cells in the spleen could participate in the host response to *P. yoelii* infection.

### Phenotypic and functional characteristics of PD-1^+^ γδT cells upon *P. yoelii* infection

**Fig. 2:**
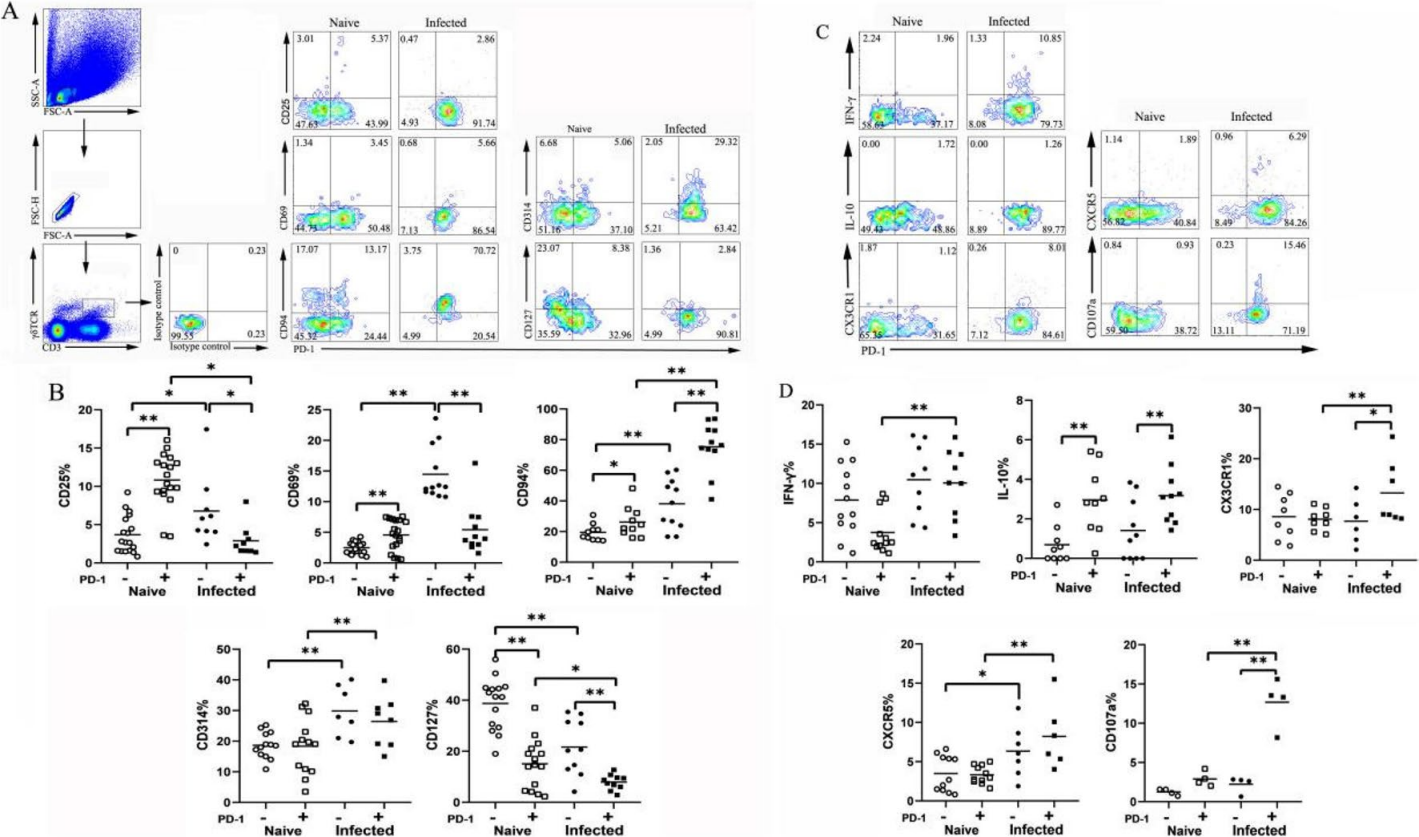
The expression of surface markers and cytokines on the PD-1^+^γδT cells. Single splenic lymphocytes were stained with fluorescent antibodies CD25, CD69, CD94, CD314, CD127. (**A**) One representative flow cytometry analysis. **B** The percentages of different surface markers were calculated. **C** Single splenic lymphocytes incubated with fluorescent antibodies: CX3CR1, CXCR5, and CD107a and then intracellularly stained with IFN-γ and IL-10. One representative flow cytometry analysis. **D** The percentages of different markers were calculated. A total of 3–5 samples were prepared for each group, and the experiments were repeated three times. One million cells from one animal were stained for the cell surface antigens. Two million cells from one animal were stained for the cell cytokines. *P < 0.05, **P < 0.01, the error bars indicate SD

To study the characteristics of PD-1^+^ γδT cells post-infection, single splenic cells were stained with fluorescent antibodies. Activation or function (CD25, CD69, CD94, CD314, and CD127), migration (CX3CR1 and CXCR5), and cytokine (IFN-γ, IL-10)-related molecules were detected by flow cytometry ( Fig. 2). PD-1-expressing γδT cells showed diminished expression of CD25, CD69, and CD127 relative to PD-1-negative γδT cells during the progression of infection (CD25, 2.88 ± 2.81% versus 6.76 ± 6.77%, *P* < 0.05; CD69, 5.42 ± 4.08% versus 14.46 ± 4.34%, *P* < 0.01; CD127, 7.91 ± 2.97% versus 21.63 ± 11.00%, *P* < 0.01). More PD-1^+^ γδT cells than PD-1^−^ γδT cells expressed CD94 (*P* < 0.01). Similarly, the proportion of CD94-expressing PD-1^+^ γδT cells increased post-infection compared to that of naïve controls (75.27 ± 16.23% versus 26.17 ± 9.95%, *P* < 0.01). In addition, compared with PD-1^+^ γδT cells from naïve mice, more of them expressed CD314 in the infected mice (*P* < 0.01). Although the proportion of IFN-γ-secreting PD-1^+^ γδT cells was increased after *P. yoelii* NSM (*P* < 0.01), no significant difference was found between the PD-1-positive or PD-1-negative γδT cells during infection (*P* > 0.05). PD-1^+^ γδT cell degranulation was determined by CD107a expression. During infection, γδT cell degranulation significantly increased in PD-1^+^ γδT cells but not in PD-1^−^ γδT cells (*P* < 0.01). In addition, the expression levels of IL-10 and CX3CR1 were significantly increased in PD-1-expressing γδT cells compared with PD-1-negative γδT cells (*P* < 0.01).

### PD-1^+^ γδT cells exhibit an effector memory phenotype

**Fig. 3:**
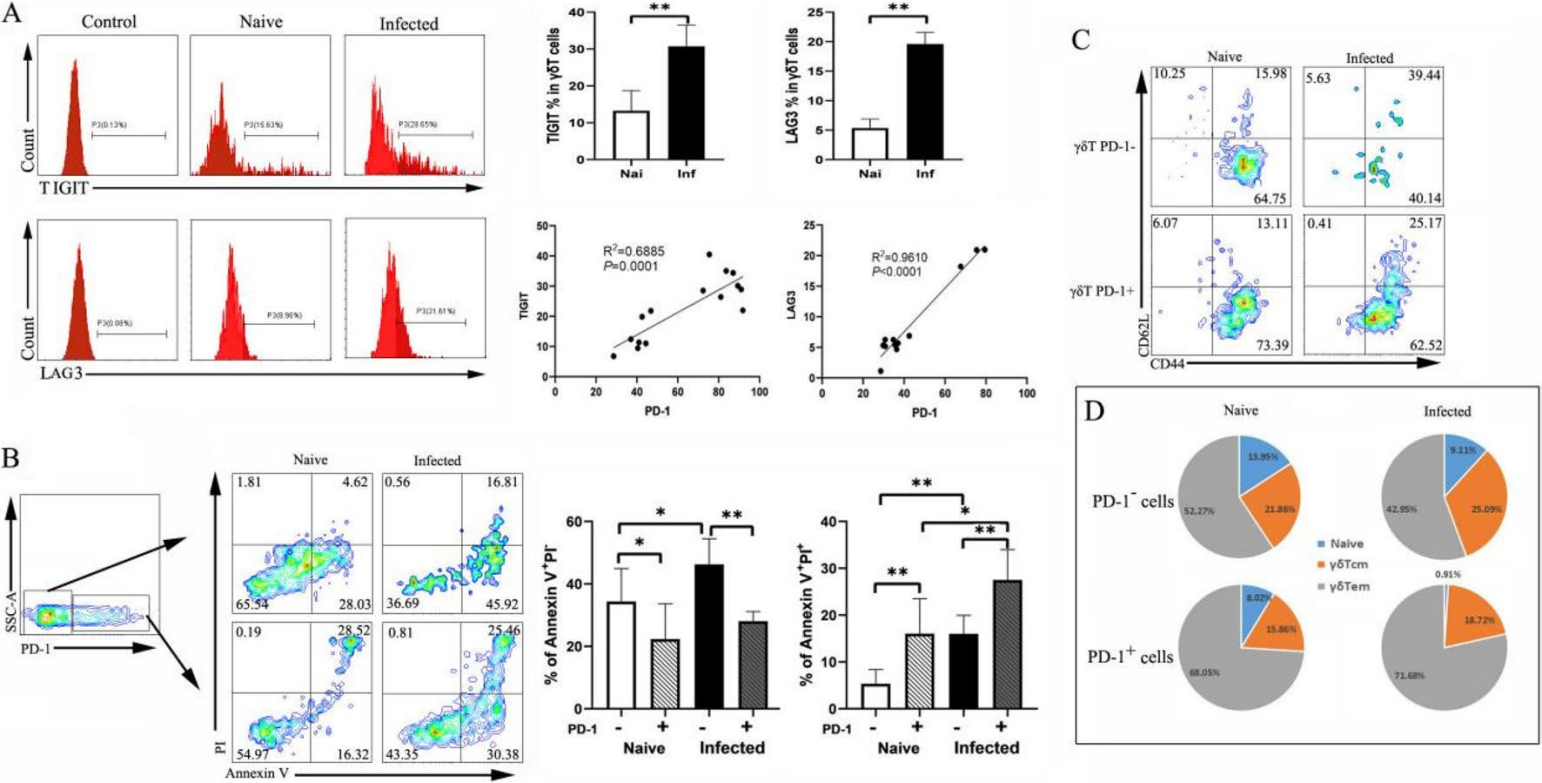
Characterization of co-expression of checkpoints, apoptosis, and memory of PD-1^+^ γδT cells. **A** γδT cells from naive and infected mice were analyzed for TIGIT and LAG-3 expression. The correlations between the expression of TIGIT and LAG-3 and PD-1 in naive and *P. yoelii* infected mice. **B** Representative contour plots showing the apoptosis of PD-1^+/−^ γδT cells from naive and infected mice (left). The percentage changes of Annexin V^+^PI^−^ and Annexin V^+^PI^+^ were calculated (right). **C** Representative contour plot to distinguish between CD62L^+^CD44^−^ (Naïve), CD62L^+^CD44^+^(CM), and CD62L^-^CD44^+^( EM) cells among PD-1^-^γδT and PD-1^+^γδT cells from naive and infected mice. **D** Frequencies of naïve γδT, γδTcm, and γδTem cells among PD-1^−^ γδT and PD-1^+^γδT cells from naive and infected mice. A total of 3–5 samples were prepared for each group, and the experiments were repeated three times. One million cells from one animal were stained for the cell surface antigens. *P < 0.05, **P < 0.01, the error bars indicate SD

We further examined the expression patterns of TIGIT and LAG-3, which were reported to be inhibitory receptors together with PD-1 expressed on immune cells. γδT cells exhibited upregulated TIGIT and LAG-3 expression after *P. yoelii* NSM infection (TIGIT, 13.24 ± 5.50% versus 30.75 ± 5.75%, *P* < 0.01; LAG-3, 5.33 ± 1.55% versus 19.61 ± 1.97%, *P* < 0.01) (Fig. 3). There were statistically significant correlations between TIGIT expression and the percentage of PD-1^+^ γδT cells and between LAG-3 expression and the percentage of PD-1^+^ γδT cells (*P* < 0.01). Studies have also shown that malarial infections cause apoptosis of immune cells. To investigate whether the level of PD-1 expression affects apoptotic cell death following *P. yoelii* NSM infection, apoptotic cell death was analyzed using the Annexin V-based flow cytometric method. As shown in Fig. 3B, more early apoptotic death was detected in PD-1-nonexpressing cells than in their PD-1-expressing counterparts, while more late apoptotic and necrotic cells were detected in PD-1-expressing cells after infection. On the basis of CD44 and CD62L expression, γδT cells can be divided into naïve γδT cells (CD62L^+^CD44^-^), central memory γδT cells (CD62L^+^CD44^+^), and effector memory T cells (CD62L^-^CD44^+^). To investigate the differentiation status of PD-1^+^ γδT cells, cells were further evaluated for CD44 and CD62L expression. A higher percentage of PD-1^+^ γδT cells (compared with PD-1^−^ γδT cells) showed effector memory features during infection (71.68 ± 8.74% versus 42.95 ± 7.77%, *P* < 0.01, Fig. 3D).

### *P. yoelii* NSM infection induces transcriptomic changes in splenic PD-1-expressing γδT cells

**Fig. 4:**
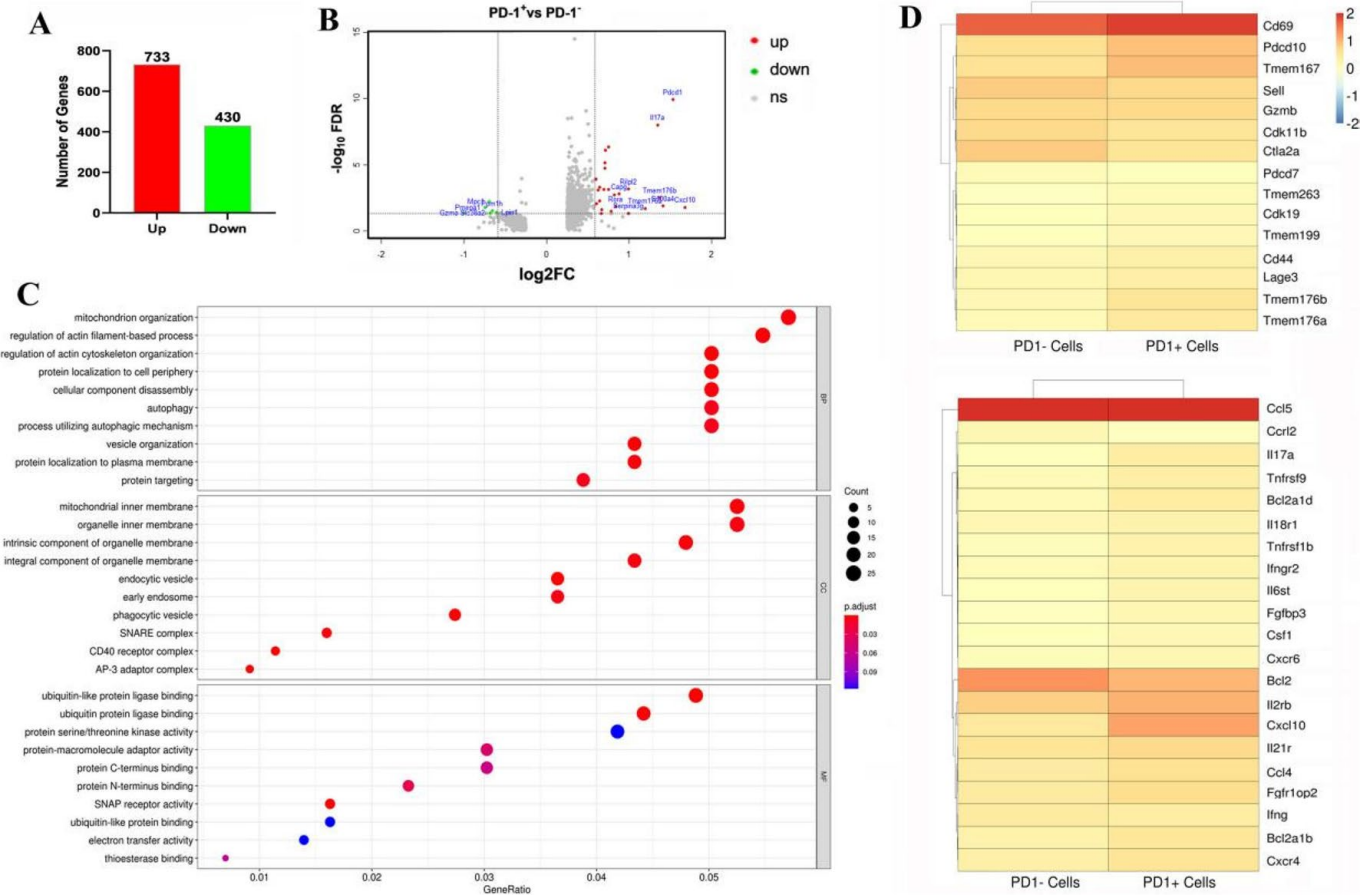
RNA-sequencing analysis of the differently expressed genes between PD-1^−/+^ γδT cells post *P. yoelii* infection. The PD-1^+^ and PD-1^−^ γδT cells from the spleens of mice infected with *P. yoeli*i (14 days post-infection) were sorted and sequenced by Single-cell RNA-sequencing. **A** Numbers of up-regulated and down-regulated genes in PD-1^+^γδT cells cells. **B** Differential gene expression was summarized in the mean difference (MD) plot of log2 expression fold-changes against the average log expressions for each gene. The differentially expressed (DE) genes relative to a fold change threshold of 1.5 are highlighted, with points colored in red and green indicating up- and down-regulated genes, respectively. **C** GO enrichment of different expressed genes. GO, Genetic Ontology; BP, biological process; CC, cellular component; MF, molecular function. **D** The heatmap for the surface molecules and cytokines expressed in PD-1^−/+^ γδT cells

To investigate the properties of PD-1-expressing γδT cells with malaria infection, the RNA expression profile was determined using single-cell RNA sequencing (10 × Genomics Chromium system). Regarding the large amount of data obtained by RNAseq, we conducted a screen of the genes in PD-1-expressing γδT cells *vs.* PD-1-nonexpressing γδT cell–infected groups. Then, we obtained 1163 DEGs: 733 were upregulated and 430 were downregulated (*P* < 0.05 or *Q* < 0.05, Fig. 4A). The differential expression multiple was screened as > 1.5, IL-17a, cxcl10, S100a4, and Rora were significantly upregulated, and Gzma, Pmepa1, and Lpin1 were significantly downregulated. To explore the potential functions of these genes, Gene Ontology enrichment analysis was performed. The results of GO analysis revealed that these differentially expressed genes were mainly involved in “autophagy” and “process utilizing autophagic mechanism”, etc. In addition, the analysis data confirmed that PD-1^+^ γδT cells expressed more surface molecules such as Pdcd10 and members of the transmembrane (TMEM) protein family (Fig. 4D). These results confirmed that cxcl10 and Il2rb were highly expressed in PD-1^+^ γδT cells and that the expression of Bcl2 was decreased (Fig. 4D).

### PD-1 expression was induced through Rorα post-*P. yoelii* infection

**Fig. 5:**
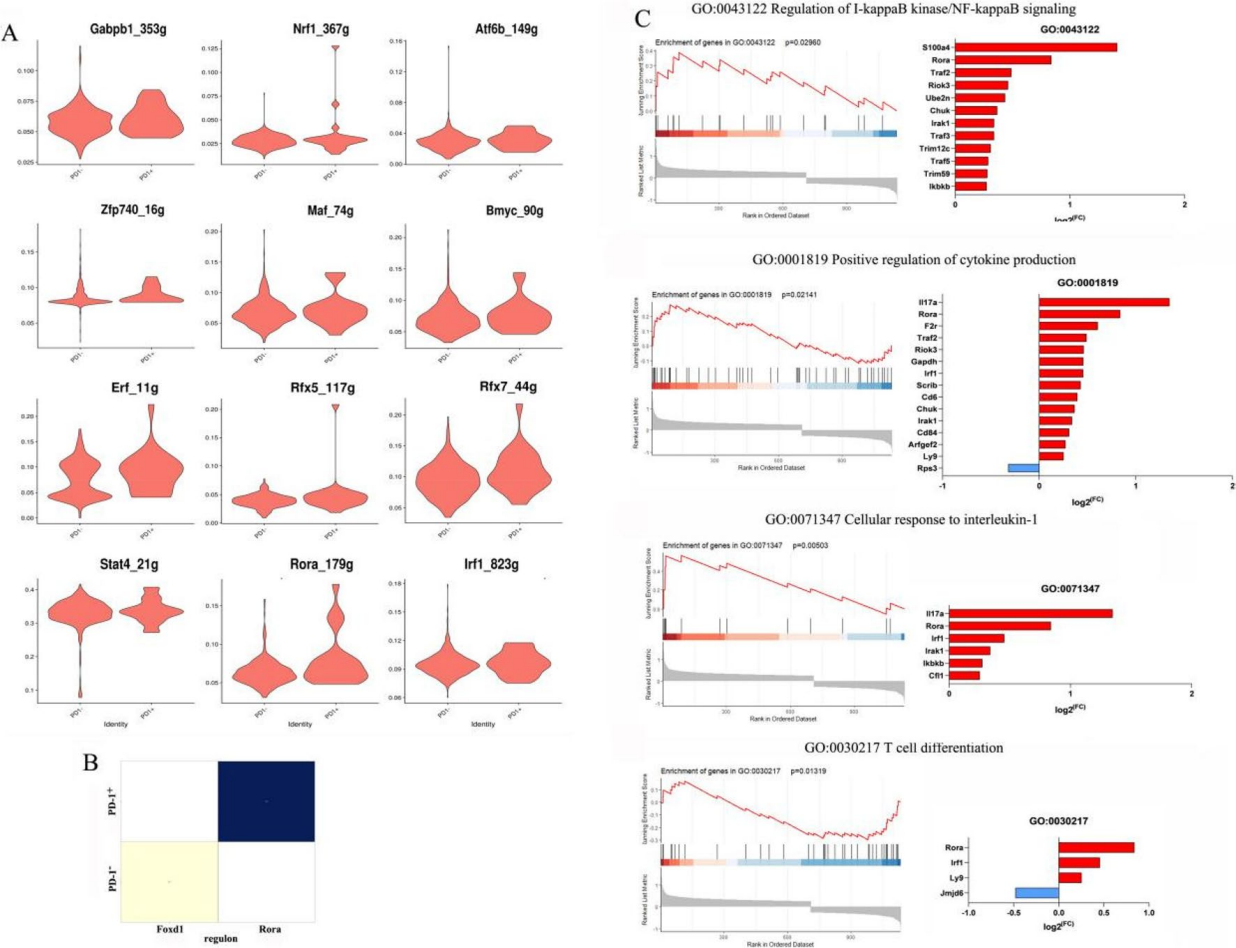
Differentially expressed transcription factors in PD-1^−/+^ γδT cells post *P. yoelii* infection. **A** The violin of the area under the curve (AUC) scores of transcription factors (TF) motifs. **B** The heatmap of the two largest differentially expressed transcription factors in PD-1^+^γδT cells and PD-1^−^γδT cells. **C** Gene-set enrichment analysis (GSEA) using the transcriptomes of PD-1^+^ γδT cells. There is a significantly enriched expression of Rora associated with the regulation of I-kappaB kinase/NF-kappaB signaling, positive regulation of cytokine, cellular response to interleukin-1, and T-cell differentiation

To further study the transcription factors (TF) that initiate PD-1 expression in γδT cells in *P. yoelii*–infected mice, expression profiles of the reported 12 transcriptional activators/repressors were compared (Fig. 5). Violin plots of the area under the curve (AUC) scores of TF motifs show the top twelve differentially expressed motifs, including Gabpb1, Nrf1, Atf6b, Zfp740, Maf, Bmyc, Erf, Rfx5, Rfx7, Stat4, Rora, and Irf1. Two transcription factors, Rora and Foxd1, showed the largest difference across all TFs in PD-1^+^ γδT cells and PD-1^−^ γδT cells, respectively. Ranked gene set enrichment analysis (GSEA) comparing PD-1-positive γδT cells to PD-1-negative γδT cells revealed that Rorα was associated with the regulation of I-kappaB kinase/NF-kappaB signaling, positive regulation of cytokine production, cellular response to interleukin-1 and T-cell differentiation. Genes driving the regulation of I-kappaB kinase/NF-kappaB signaling were S100 calcium-binding protein A4, tumor necrosis factor receptor-associated factors (TRAF), serine/threonine-protein kinase RIO3 (RIOK3), ubiquitin-conjugating enzyme E2 N (UBE2N), and so on. There were also genes that were enriched for the acellular response to interleukin-1, as shown by the upregulation of cytokine gene (Il17a), interferon regulatory factor-1 (Irf1), interleukin-1 receptor-associated kinase 1 (Irak1), and inhibitor of kappa-light polypeptide gene enhancer in B-cell kinase beta (IkBKb).

### RORα blockade upregulated the NF-κB pathway

**Fig. 6:**
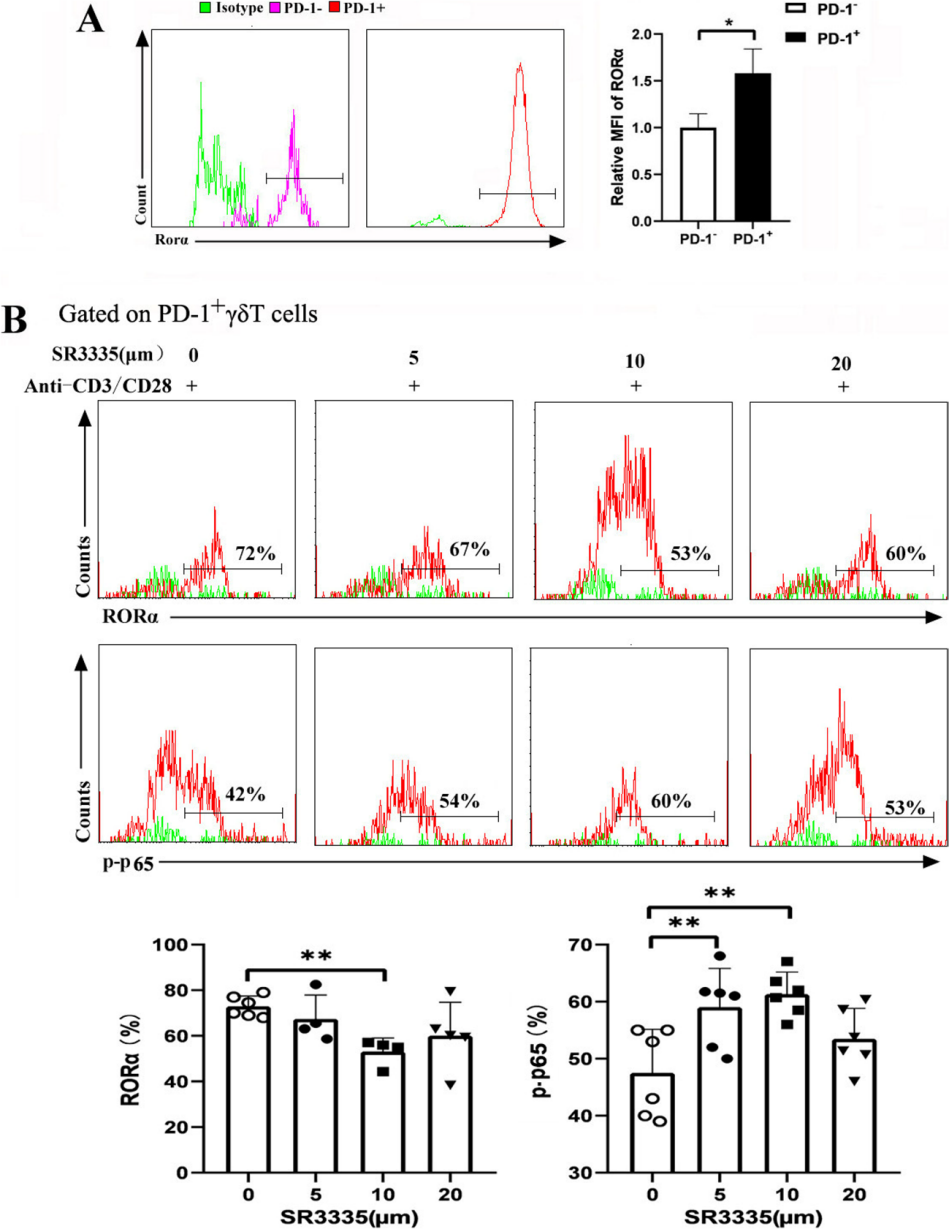
RORα inhibitors possessed the ability to increase the expression of p-NF-κβ. Female C57BL/6 mice were infected with *P. yoelii*. Splenocytes were separated (12–14 days post-infection) and then stained with monoclonal antibodies against mouse CD3, γδTCR, PD-1 for flow cytometry analysis. **A** The expressions of RORα in PD-1^+/−^γδT cells were measured by flow cytometry (left). The numbers represent the relative MFI of stained RORα protein (right). **B** Normal female C57BL/6 mice were sacrificed. Splenocytes were treated with SR3335 at doses of 5, 10, and 20 μm for 48 h. Intranuclear amount of p-p65 were measured. The counts of RORα and p-p65 in PD-1^+^γδT cells were analyzed by flow cytometry and the percentages were calculated. *P < 0.05, **P < 0.01, the error bars indicate SD

According to the above results, RORα was speculated to be the key transcription factor that induces PD-1 expression after *P. yoelii* infection. The mean fluorescence intensity (MFI) of RORα was higher in PD-1^+^ γδT cells than in PD-1^−^γδT cells in the infected mice (Fig. 6A). This result indicated that RORα has the ability to downregulate NF-κB signaling. The NF-κB family consists of p65/Rel A, p50/NF-κB1, p52, Rel B, and c-Rel. Among them, the p50-p65 heterodimer is the most abundant. The specific antagonist small molecule SR3335, which enhanced NF-κB luciferase activity, was used to inhibit RORα (Fig. 6B). We examined whether the expression of p-p65 was altered by SR3335 treatment. Mice were infected with *P. yoelii* 12 to 14 days later, and the spleens were removed. Single cells were treated with SR3335 at doses of 5, 10, and 20 μM for 2 days. When the splenic cells were treated with 10 μM SR3335 and anti-CD3/CD28, the expression of RORα was significantly downregulated compared with that of cells treated only with anti-CD3/CD28 (*P* < 0.01). Furthermore, the suppression of RORα led to the upregulation of NF-κB in PD-1^+^ γδT cells after 10 μM SR3335 treatment for 48 h (*P* < 0.01). Taken together, these results show that RORα regulates the effector responses of PD-1^+^ γδT cells in an NF-κB-dependent manner.

## Discussion

γδT cells, a unique immune population, undergo dramatic expansion during acute *Plasmodium* infections and have been shown to have anti-parasitic functions in both mice and humans [3, 33]. Our previous results also indicated that γδT cells accumulate in the spleen, lung, liver, mesenteric lymph nodes, and peripheral blood mononuclear cells of infected mice and may play a role in the process of host anti-*Plasmodium* infection. However, less is known about the prevalence and function of PD-1^+^ γδT cells in the context of malaria. In this study, we found that *Plasmodium* parasites upregulate PD-1 on CD4^+^ T cells, CD8^+^ T cells, and γδ T cells. Furthermore, we investigated the dynamic change in PD-1^+^ γδT cells from different stages of *Plasmodium yoelii* infection in mice (Fig. 1C). The results demonstrated that the proportion of PD-1^+^ γδT cells in the spleen increased continuously during the early stage, peaked at 12–20 days, and decreased at 24 days after infection. Overall, this may correlate with the intensity of the pathogenic immune response that is induced by the *Plasmodium yoelii* parasite.

It was reported that PD-1 expression on NK cells in malaria-exposed individuals is associated with diminished natural cytotoxicity and enhanced antibody-dependent cellular cytotoxicity [34]. Additionally, PD-1^+^ CD4^+^ T cells appeared to be more activated and could secrete more cytokines to regulate the host’s immune responses against malaria, as emphasized by Wei H et al [26]. As shown in Fig. 2, PD-1-expressing γδT cells expressed less CD25, CD69, and CD127 but more CD94 than PD-1-negative γδT cells. CD25 and CD69 are proposed to be expressed on both activated T cells and some regulatory T cells [35]. This finding may reveal that the increased distinct inhibitory receptors and diminished distinct activated receptors were associated with PD-1-expressing γδT cell exhaustion. In our study, γδT cell degranulation was quantified by intracellular CD107a expression. Moreover, CX3CR1, a chemokine receptor, is mainly expressed on cytotoxic effector lymphocytes, which influence the migration profile and functionality of immune cells [36]. In comparison to PD-1^−^ γδT cells, we observed a significant increase in degranulation and the expression of CX3CR1 in PD-1^+^ γδT cells. PD-1^+^ γδ T cells may inhibit parasite replication by targeting and killing extracellular merozoites or intracellular late-stage parasites through degranulation and have migratory capacity. IL-10, a key cytokine, has an essential regulatory function in controlling the inflammatory response and preventing or ameliorating a wide range of clinical manifestations during malaria [37]. It is possible that IL-10 promotes parasite persistence by decreasing the production of Th1 cytokines such as IFN-γ, IL-12, and TNF-α. These results confirm that PD-1^+^ γδT cells exhibit a special phenotype, with limited activation but no inhibition of cytolytic function during infection, consistent with previous studies suggesting that γδT cells may paradoxically contribute to both protection and pathology during *Plasmodium* infection [38–40].

It has long been observed that PD-1, LAG-3, CTLA-4, TIGIT, and TIM-3 are negatively correlated with polyfunctionality [20]. It is also interesting to note a positive correlation between the expression of PD-1 on γδT cells and LAG3 and TIGIT (Fig. 3A). Importantly, immune checkpoints have distinct mechanisms and nonredundant roles, emphasizing the complexity of the regulation of γδT cell responses. Our results showed that PD-1 caused late apoptotic death of γδT cells rather than early apoptotic death during malarial infections. Upon exposure to parasite antigens, some effector cells will further differentiate into memory T cells, which can provide different signals than their naïve and effector counterparts and proliferate faster in response to antigen exposure [41]. Furthermore, memory T cells can be subdivided into central memory T cells (TCM) and effector memory T cells (TEM) [42]. γδT cells are thought to follow a stepwise, progressive shift from naïve T cells to effector T cells (TEFF) cells, then TCM cells, and eventually TEM cells based on antigen recognition, activation, and the presence of cytokines in mice and humans [43, 44]. Our results showed that increased expression of PD-1 was predominantly observed on effector memory γδ T cells. Previous studies revealed that the interplay of multiple immune checkpoints and their ligands occurs at different stages of cell activation and function [20, 45]. PD-1 expression is upregulated on activated T cells, and ligation of PD-1 with PD-L1 or PD-L2 predominantly occurs in the periphery, leading to suppression of activated T cells at the effector phase [46]. PD-1-expressing γδT cells exhibit hallmarks of both exhausted and effector memory and co-expressed LAG3 and TIGIT.

Single-cell RNA-seq was applied to dissect cell heterogeneity (Fig. 4). We discovered that the gene profile in PD-1-expressing γδT cells was significantly different from that in PD-1-negative γδT cells from infected mice. Notably, differentially expressed genes in PD-1-expressing γδT cells were positively correlated with autophagy and processes utilizing autophagic mechanisms. Autophagy, a process by which cells recycle their cytoplasmic contents in lysosomes, plays important roles in the elimination of pathogens, control of inflammation, and adaptive immunity [47]. Recent studies have delineated the mechanism underlying autophagy and the intricate involvement of the PD-L1/PD1 axis in multiple diseases, including ovarian cancer, melanoma, and myocarditis [48, 49]. One study reported that anti-PD-L1 antibody promotes protective immunity against visceral leishmaniasis via autophagy inhibition [50]. Taken together, our results are consistent with previous reports demonstrating a positive relationship between the PD1/PDL-1 pathway and autophagy during infection. Furthermore, the PD-1-expressing γδT cell population displayed significant increases in the expression of genes associated with chemokines and receptors (cxcl10, cxcr4, and cxcr6). This result suggested that the migration of PD-1-expressing γδT cells toward the source of chemokines was guided by chemokines and their receptors underlying the pathogenesis of *Plasmodium* infection.

Our results confirmed that Rora was the key transcription factor for PD-1-expressing γδT cells after *P. yoelii* infection (Fig. 5). RORα, a member of the orphan nuclear receptor (ONR) family, plays a critical role in the regulation of inflammation during migraine, cancer, and multiple sclerosis [51–53]. Although various functions of RORα as a negative regulator of the NF-κB signaling pathway have been shown [54, 55], the molecular mechanisms associated with antimalarial immunity remain to be determined. Consistent with this finding, our gene-set enrichment analysis (GSEA) illustrated the enrichment of RORα and other genes in the regulation of I-kappaB kinase/NF-kappaB signaling. SR3335, a RORα-specific inhibitor, was employed to explore the effect of RORα in regulating NF-kappaB signaling downstream. Compared with vehicle treatment, SR3335 treatment ex vivo significantly increased the expression of p-p65 at concentrations of 5 μM and 10 μM (Fig. 6). Accordingly, this study identifies that RORα may be crucial during the progression of malaria infection in PD-1-expressing γδT cells by attenuating NF-κB transcriptional activity.

In conclusion, this study suggests that RORα-mediated attenuation of NF-κB signaling may be fundamental for PD-1-expressing γδT cells to modulate host immune responses during malaria infection.

## Data Availability

The datasets presented in this study can be found in online repositories. The names of the repository/repositories and accession number(s) can be found below: bioproject/,PRJNA702594 and PRJNA702837, https://www.st-va.ncbi.nlm.nih.gov/.

## References

[1] Xie H, Xie S, Wang M (2021). Properties and Roles of γδT Cells in Plasmodium yoelii nigeriensis NSM Infected C57BL/6 Mice. Front Cell Infect Microbiol..

[2] Daily JP, Minuti A, Khan N (2022). Diagnosis, treatment, and prevention of malaria in the US: a review. JAMA..

[3] Pamplona A, Silva-Santos B (2021). γδ T cells in malaria: a double-edged sword. FEBS J..

[4] Ortiz-Ruiz A, Postigo M, Gil-Casanova S (2018). Plasmodium species differentiation by non-expert on-line volunteers for remote malaria field diagnosis. Malar J..

[5] Yahata K, Treeck M, Culleton R, Gilberger TW, Kaneko O (2012). Time-lapse imaging of red blood cell invasion by the rodent malaria parasite Plasmodium yoelii. PLoS One..

[6] Ramiro RS, Reece SE, Obbard DJ (2012). Molecular evolution and phylogenetics of rodent malaria parasites. BMC Evol Biol..

[7] Kordes M, Ormond L, Rausch S, Matuschewski K, Hafalla J (2022). TLR9 signalling inhibits Plasmodium liver infection by macrophage activation. Eur J Immunol..

[8] Cowman AF, Healer J, Marapana D, Marsh K (2016). Malaria: biology and disease. Cell..

[9] Hviid L, Lopez-Perez M, Larsen MD, Vidarsson G (2022). No sweet deal: the antibody-mediated immune response to malaria. Trends Parasitol..

[10] Van Braeckel-Budimir N, Kurup SP, Harty JT (2016). Regulatory issues in immunity to liver and blood-stage malaria. Curr Opin Immunol..

[11] Bhat SA, Vedpathak DM, Chiplunkar SV (2018). Checkpoint blockade rescues the repressive effect of histone deacetylases inhibitors on γδ T cell function. Front Immunol..

[12] Khairallah C, Chu TH, Sheridan BS (2018). Tissue adaptations of memory and tissue-resident gamma delta T cells. Front Immunol..

[13] Brandes M, Willimann K, Bioley G (2009). Cross-presenting human gammadelta T cells induce robust CD8+ alphabeta T cell responses. Proc Natl Acad Sci USA..

[14] Hernández-Castañeda MA, Happ K, Cattalani F (2020). γδ T cells kill Plasmodium falciparum in a granzyme- and granulysin-dependent mechanism during the late blood stage. J Immunol..

[15] Jagannathan P, Lutwama F, Boyle MJ (2017). Vδ2+ T cell response to malaria correlates with protection from infection but is attenuated with repeated exposure. Sci Rep..

[16] Pauken KE, Wherry EJ (2015). Overcoming T cell exhaustion in infection and cancer. Trends Immunol..

[17] Wykes MN, Zhou YH, Liu XQ, Good MF (2005). Plasmodium yoelii can ablate vaccine-induced long-term protection in mice. J Immunol..

[18] Pierce SK, Miller LH (2009). World Malaria Day 2009: what malaria knows about the immune system that immunologists still do not. J Immunol..

[19] Wykes MN, Lewin SR (2018). Immune checkpoint blockade in infectious diseases. Nat Rev Immunol..

[20] Dyck L, Mills K (2017). Immune checkpoints and their inhibition in cancer and infectious diseases. Eur J Immunol..

[21] Wherry EJ, Ha SJ, Kaech SM (2007). Molecular signature of CD8+ T cell exhaustion during chronic viral infection. Immunity..

[22] Furtado R, Chorro L, Zimmerman N (2020). Blockade of LAG-3 in PD-L1-deficient mice enhances clearance of blood stage malaria independent of humoral responses. Front Immunol..

[23] Wykes MN, Horne-Debets JM, Leow CY, Karunarathne DS (2014). Malaria drives T cells to exhaustion. Front Microbiol..

[24] Chandele A, Mukerjee P, Das G, Ahmed R, Chauhan VS (2011). Phenotypic and functional profiling of malaria-induced CD8 and CD4 T cells during blood-stage infection with Plasmodium yoelii. Immunology..

[25] Illingworth J, Butler NS, Roetynck S (2013). Chronic exposure to Plasmodium falciparum is associated with phenotypic evidence of B and T cell exhaustion. J Immunol..

[26] Wei H, Xie A, Li J (2022). PD-1+ CD4 T cell immune response is mediated by HIF-1α/NFATc1 pathway after *P. yoelii* infection. Front Immunol..

[27] LaFleur MW, Nguyen TH, Coxe MA (2019). PTPN2 regulates the generation of exhausted CD8+ T cell subpopulations and restrains tumor immunity. Nat Immunol..

[28] Khan O, Giles JR, McDonald S (2019). TOX transcriptionally and epigenetically programs CD8+ T cell exhaustion. Nature..

[29] Chen D, Guo Y, Jiang J (2022). γδ T cell exhaustion: opportunities for intervention. J Leukoc Biol..

[30] Chi Z, Lu Y, Yang Y, Li B, Lu P (2021). Transcriptional and epigenetic regulation of PD-1 expression. Cell Mol Life Sci..

[31] Wherry EJ, Kurachi M (2015). Molecular and cellular insights into T cell exhaustion. Nat Rev Immunol..

[32] Lino C, Barros-Martins J, Oberdörfer L, Walzer T, Prinz I (2017). Eomes expression reports the progressive differentiation of IFN-γ-producing Th1-like γδ T cells. Eur J Immunol..

[33] Ribot JC, deBarros A, Pang DJ (2009). CD27 is a thymic determinant of the balance between interferon-gamma- and interleukin 17-producing gammadelta T cell subsets. Nat Immunol..

[34] Moebius J, Guha R, Peterson M (2020). PD-1 Expression on NK Cells in malaria-exposed individuals is associated with diminished natural cytotoxicity and enhanced antibody-dependent cellular cytotoxicity. Infect Immun.

[35] Xie H, Chen D, Li L (2014). Immune response of γδT cells in Schistosome japonicum-infected C57BL/6 mouse liver. Parasite Immunol..

[36] Familiar-Macedo D, Amancio Paiva I, Badolato-Corrêa da Silva J, et al. Evaluation of the expression of CCR5 and CX3CR1 receptors and correlation with the functionality of T cells in women infected with ZIKV during pregnancy. Viruses. 2021;13(2):1–18 10.3390/v13020191 PMC7912595 33525328

[37] Freitas do Rosario AP, Langhorne J. (2012). T cell-derived IL-10 and its impact on the regulation of host responses during malaria. Int J Parasitol..

[38] Jagannathan P, Kim CC, Greenhouse B (2014). Loss and dysfunction of Vδ2^+^ γδ T cells are associated with clinical tolerance to malaria. Sci Transl Med..

[39] Costa G, Loizon S, Guenot M (2011). Control of Plasmodium falciparum erythrocytic cycle: γδ T cells target the red blood cell-invasive merozoites. Blood..

[40] Stanisic DI, Cutts J, Eriksson E (2014). γδ T cells and CD14+ monocytes are predominant cellular sources of cytokines and chemokines associated with severe malaria. J Infect Dis..

[41] Comeau K, Paradis P, Schiffrin EL (2020). Human and murine memory γδ T cells: evidence for acquired immune memory in bacterial and viral infections and autoimmunity. Cell Immunol..

[42] Kumarasingha R, Ioannidis LJ, Abeysekera W (2020). Transcriptional memory-like imprints and enhanced functional activity in γδ T cells following resolution of malaria infection. Front Immunol..

[43] Golubovskaya V, Wu L (2016). Different subsets of T cells, memory, effector functions, and CAR-T immunotherapy. Cancers (Basel).

[44] Mahnke YD, Brodie TM, Sallusto F, Roederer M, Lugli E (2013). The who’s who of T-cell differentiation: human memory T-cell subsets. Eur J Immunol..

[45] Anderson AC, Joller N, Kuchroo VK (2016). Lag-3, Tim-3, and TIGIT: Co-inhibitory receptors with specialized functions in immune regulation. Immunity..

[46] Topalian SL, Drake CG, Pardoll DM (2012). Targeting the PD-1/B7-H1(PD-L1) pathway to activate anti-tumor immunity. Curr Opin Immunol..

[47] Deretic V, Saitoh T, Akira S (2013). Autophagy in infection, inflammation and immunity. Nat Rev Immunol..

[48] Clark CA, Gupta HB, Sareddy G (2016). Tumor-Intrinsic PD-L1 signals regulate cell growth, pathogenesis, and autophagy in ovarian cancer and melanoma. Cancer Res.

[49] Liu YX, Song YJ, Liu XH (2022). PD-1 inhibitor induces myocarditis by reducing regulatory T cells, activating inflammatory responses, promoting myocardial apoptosis and autophagy. Cytokine..

[50] Habib S, El Andaloussi A, Elmasry K (2018). PDL-1 blockade prevents T Cell exhaustion, inhibits autophagy, and promotes clearance of Leishmania donovani. Infect Immun..

[51] Farahani S, Solgi L, Bayat S (2020). RAR-related orphan receptor A: one gene with multiple functions related to migraine. CNS Neurosci Ther..

[52] Cai X, Lin M, Cao S, Liu Y, Lin N (2018). The association of RAR-related orphan receptor A (RORA) gene polymorphisms with the risk of asthma. Ann Hum Genet..

[53] Kim K, Lee JM, Yu YS (2017). RORα2 requires LSD1 to enhance tumor progression in breast cancer. Sci Rep..

[54] Oh SK, Kim D, Kim K (2019). RORα is crucial for attenuated inflammatory response to maintain intestinal homeostasis. Proc Natl Acad Sci USA..

[55] Lee IK, Song H, Kim H (2020). RORα Regulates cholesterol metabolism of CD8+ T cells for anticancer immunity. Cancers (Basel)..

