# Correction to: Characteristics of splenic PD-1^+^ γδT cells in *Plasmodium yoelii nigeriensis* infection

**DOI:** 10.1007/s12026-024-09469-6

**Published:** 2024-02-29

**Authors:** Dianhui Chen, Feng Mo, Meiling Liu, Lin Liu, Junmin Xing, Wei Xiao, Yumei Gong, Shanni Tang, Zhengrong Tan, Guikuan Liang, Hongyan Xie, Jun Huang, Juan Shen, Xingfei Pan

**Affiliations:** 1 Department of Infectious Diseases, Guangdong Provincial Key Laboratory of Major Obstetric Diseases, Guangdong Provincial Clinical Research Center for Obstetrics and Gynecology, The Third Affiliated Hospital of Guangzhou Medical University, Guangzhou, China; 2 Clinical Laboratory, The Third Affiliated Hospital of Guangzhou Medical University, Guangzhou, 510150 China; 3 China Sino-French Hofmann Institute, Department of Basic Medical Science, Guangzhou Medical University, Guangzhou, 511436 China; 4 State Key Laboratory of Respiratory Disease, National Clinical Research Center for Respiratory Disease, Guangzhou, China; 5 Kingmed School of Laboratory Medicine, Guangzhou Medical University, Guangzhou, Guangdong 510182 People’s Republic of China

**Correction to: Immunologic Research**

10.1007/s12026-023-09441-w

The originally published version of this article contained mistakes in the first affiliation and the authors would like to correct them.

The affiliation of the authors Dianhui Chen, Feng Mo, Shanni Tang and Xingfei Pan were incorrectly given as “Department of Infectious Diseases, The Third Afliated Hospital of Guangzhou Medical University, Guangzhou 510150, China.” The correct affiliation is presented below.

^1^Department of Infectious Diseases, Guangdong Provincial Key Laboratory of Major Obstetric Diseases; Guangdong Provincial Clinical Research Center for Obstetrics and Gynecology; The Third Affiliated Hospital of Guangzhou Medical University, Guangzhou, China

The original article has been corrected.

